# Beneficial Metabolic Effects of Rapamycin Are Associated with Enhanced Regulatory Cells in Diet-Induced Obese Mice

**DOI:** 10.1371/journal.pone.0092684

**Published:** 2014-04-07

**Authors:** Kassem Makki, Solenne Taront, Olivier Molendi-Coste, Emmanuel Bouchaert, Bernadette Neve, Elodie Eury, Stéphane Lobbens, Myriam Labalette, Hélène Duez, Bart Staels, David Dombrowicz, Philippe Froguel, Isabelle Wolowczuk

**Affiliations:** 1 Centre National de la Recherche Scientifique (CNRS), Unité Mixte de Recherche (UMR)8199, Lille Pasteur Institute, Lille, France; 2 Lille 2 University, Lille, France; 3 European Genomic Institute for Diabetes (EGID), Lille, France; 4 Institut National de la Santé et de la Recherche Médicale (Inserm), UMR1011, Lille Pasteur Institute, Lille, France; 5 Immunology Institute, Centre Hospitalier Régional Universitaire (CHRU) Lille and Equipe d'Accueil (EA)2686, Lille 2 University, Lille, France; 6 Department of Genomics of Common Disease, School of Public Health, Imperial College London, London, United Kingdom; University Dresden, Germany

## Abstract

The “mechanistic target of rapamycin” (mTOR) is a central controller of growth, proliferation and/or motility of various cell-types ranging from adipocytes to immune cells, thereby linking metabolism and immunity. mTOR signaling is overactivated in obesity, promoting inflammation and insulin resistance. Therefore, great interest exists in the development of mTOR inhibitors as therapeutic drugs for obesity or diabetes. However, despite a plethora of studies characterizing the metabolic consequences of mTOR inhibition in rodent models, its impact on immune changes associated with the obese condition has never been questioned so far. To address this, we used a mouse model of high-fat diet (HFD)-fed mice with and without pharmacologic mTOR inhibition by rapamycin. Rapamycin was weekly administrated to HFD-fed C57BL/6 mice for 22 weeks. Metabolic effects were determined by glucose and insulin tolerance tests and by indirect calorimetry measures of energy expenditure. Inflammatory response and immune cell populations were characterized in blood, adipose tissue and liver. In parallel, the activities of both mTOR complexes (*e. g.* mTORC1 and mTORC2) were determined in adipose tissue, muscle and liver. We show that rapamycin-treated mice are leaner, have enhanced energy expenditure and are protected against insulin resistance. These beneficial metabolic effects of rapamycin were associated to significant changes of the inflammatory profiles of both adipose tissue and liver. Importantly, immune cells with regulatory functions such as regulatory T-cells (Tregs) and myeloid-derived suppressor cells (MDSCs) were increased in adipose tissue. These rapamycin-triggered metabolic and immune effects resulted from mTORC1 inhibition whilst mTORC2 activity was intact. Taken together, our results reinforce the notion that controlling immune regulatory cells in metabolic tissues is crucial to maintain a proper metabolic status and, more generally, comfort the need to search for novel pharmacological inhibitors of the mTOR signaling pathway to prevent and/or treat metabolic diseases.

## Introduction

The mechanistic (formerly mammalian) target of rapamycin (mTOR) is a highly conserved serine-threonine kinase that regulates cell size, survival and proliferation in response to amino acids, growth factors, nutrients and cellular energy status [1]. mTOR signaling pathway is constitutively activated in obesity, leading to insulin resistance [2], [3]. Therefore, the mTOR inhibitor rapamycin, an FDA-approved drug for patients with organ transplant [4] has been considered for treatment of metabolic disorders.

mTOR exists in two distinct functional complexes called mTOR complex-1 (mTORC1 or Raptor) and mTOR complex-2 (mTORC2 or Rictor) [5] that differ regarding their sensitivity to rapamycin. mTORC1 is highly sensitive to the drug whilst only daily and chronic exposure to rapamycin can inhibit mTORC2 activity [6], [7].

Conditional knockout of mTOR in the mouse model revealed its role in key functions of several metabolic tissues such as glycogen synthesis in muscle [8], ketogenesis and lipogenesis in the liver [9], [10] and adipogenesis in the adipose tissue [11], [12]. In obese animal models, treatment with rapamycin can reduce adiposity [13], [14]. However, how rapamycin precisely impacts on energy homeostasis is still an open question since, depending on experimental animal models (rat, mouse) or procedures (dose, route and frequency of rapamycin administration), either protective or detrimental effects were observed [13], [15]–[18].

Recent studies revealed that mTOR is also a central regulator of innate and adaptive immune responses; thus connecting metabolism and immunity. Specifically, mTOR controls the differentiation, activation and function of monocytes and macrophages as well as of B -cells and CD4 and CD8 T -cells [19], [20]. Furthermore, mTOR inhibition promotes the generation of CD4^+^ FoxP3^+^ regulatory T cells (Tregs) both *in vitro* and *in vivo* [21]–[23].

Importantly, obesity is associated with profound immune dysfunctions [24]–[26] as well as with chronic, low-grade inflammation that predisposes to the development of systemic insulin resistance [27]–[30]. Indeed, the expanding white adipose tissue (WAT) develops a chronic inflammatory response, largely resulting from increased macrophage infiltration combined with depletion of anti-inflammatory Tregs [31], [32]. Beside Tregs, a group of immature myeloid cells characterized by the co-expression of the surface markers CD11b and Gr-1 (a composite epitope between the Ly6C and Ly6G antigens) was lately reported to counter inflammation during obesity [33]. These myeloid-derived suppressor cells (MDSCs) consist of two major populations depending on Ly6C or Ly6G expression: granulocytic MDSCs (G-MDSCs), which are CD11b^+^ Ly6G^+^ Ly6C^med^, and monocytic MDSCs (M-MDSCs) which are CD11b^+^ Ly6G^−^ Ly6C^hi^ [34], [35]. In obese mice, depletion of MDSCs using Gr1-specific antibody increases insulin resistance and glucose intolerance whilst adoptive transfer of MDSCs improves metabolic parameters [33]. Therefore, maintaining and/or increasing adipose regulatory cells (*e.g.* Tregs and/or MDSCs) is crucial for preserving the insulin sensitive status [33], [36], [37].

Despite the potent immunoregulatory properties of rapamycin [38], the immunological changes associated with its administration to obese mice have never been investigated so far. Therefore, in the current study we assessed the metabolic and immunologic consequences of weekly administration of rapamycin (2 mg/kg) to HFD-fed C57BL/6 mice (the inbred mouse strain that is the most commonly used for metabolic studies) for a period of 22 weeks. We show that, while increasing systemic and adipose inflammation, rapamycin treatment alleviates liver inflammation and, thus, ameliorates the general metabolic status of obese mice. The beneficial metabolic effects of rapamycin were associated with a remodeling of adipose tissue and liver cellular composition with increased numbers of regulatory cells such as Tregs and MDSCs. At the molecular level, rapamycin treatment inhibited mTORC1 activity whilst that of mTORC2 was likely not affected. Although mechanisms through which rapamycin impacts on regulatory cells is still elusive, the present study however extends the concept that targeting the mTOR signaling pathway deserves interest in the treatment of metabolic diseases.

## Materials and Methods

### Animals and Diet and Drug Treatment

C57BL/6JRj mice were obtained from Janvier Laboratory (Le Genest-St-Isle, France). Six-week-old mice (male and female) were fed *ad libitum* with a high-fat diet (HFD; D12492, Research Diets, New Brunswick, NJ, USA) containing 60 kcal% fat. After 5 weeks of diets, mice were randomly divided into 2 groups. One group of mice was intraperitoneally (i.p.) injected with rapamycin (Rapa; 2 mg/kg body weight; LC Laboratories, Woburn, MA, USA) diluted in a vehicle solution (Ve; sterile 10% PEG400/8% ethanol, followed by an equal volume of sterile 10% Tween 80) as described in [13], once a week. The second sub-group received the corresponding volume of the Ve solution.

After 22 weeks of rapamycin (or Ve) treatment, animals were sacrificed by direct cervical dislocation to avoid the effect of anesthesia on lipid metabolism. Then, tissues (*i.e.* blood, liver, perigonadal white adipose tissue (WAT) and interscapular brown adipose tissue (BAT)) were harvested. Sera were kept at −80°C until use. For histological analysis, liver, WAT and BAT were fixed (detailed below) and maintained at 4°C. For expression studies, tissue samples were snap-frozen and stored at −80°C. Interscapular BAT was carefully dissected to avoid the muscle closely associated with it.

### Metabolic Phenotyping

*Body weight and food intake* were measured weekly. Food consumption was estimated by subtracting the amount of remaining food from that of food supplied. Spilled food pellets were carefully collected to ensure the accuracy of food intake measurements.

For *indirect calorimetry*, animals were housed individually in metabolic cages (Oxylet, Panlab, Barcelona, Spain) and acclimatized for 12 hours before the beginning of the analysis. O_2_ consumption (Vo_2_) and CO_2_ production (Vco_2_) were measured during 36 h. Energy expenditure was calculated as described [39].

*Basal core body temperature* was determined using a Thermalert temperature monitor (Physitemp, Clifton, NJ, USA).

For *glucose tolerance tests (GTT)*, animals were fasted for 6 hours before being i.p. injected with D-glucose (1 g/kg body weight; Sigma-Aldrich, Lyon, France). Glucose levels were measured by tail-tip bleeding with an automatic glucometer (ACCU-CHEK Performa, Roche, Mannheim, Germany) before injection and 15, 30, 60 and 180 minutes after glucose administration.

For *insulin tolerance tests* (ITT), animals were fasted for 6 hours before being i.p. injected with insulin (0.75 IU/kg body weight; Sigma-Aldrich). Blood glucose levels were measured before and 15, 30, 45, 60 and 75 minutes after insulin injection.

*Quantification of ketone bodies, adiponectin, leptin and insulin blood levels at sacrifice.*

Ketone bodies levels were determined in the sera of 12-hours fasted mice using beta Hydroxybutyrate (beta HB) Assay Kit (ab83390, Cambridge, UK). Adiponectin, leptin and insulin levels were measured in the sera of 6-hours fasted mice using specific ELISA kits (respectively; Quantikine Mouse Adiponectin/Acrp30 and Quantikine Mouse Leptin (R&D Systems, Minneapolis, MN, USA) and Ultrasensitive Mouse Insulin ELISA (Mercodia, Uppsala, SWE).

### Immunologic Phenotyping

#### Preparation of blood and stromal vascular fraction (SVF) cells

Blood was collected on EDTA (0.5 M). After erythrocyte lysis, aliquots of 10^6^ cells were put into 96-well microtiter plates for staining (see below).

Visceral WAT was harvested, minced and incubated for 1 hour at 37°C in digestion medium (Dulbecco's Modified Eagle Medium (DMEM) F-12; GIBCO, Life Technologies; Saint Aubin, France) supplemented with BSA (1%; Sigma-Aldrich), Gentamycin (1%; GIBCO; Life Technologies) and type I collagenase (1.5 mg/ml; Sigma-Aldrich). Cells from the stromal vascular fraction (SVF) were then collected after centrifugation for 15 min at 250 *g*. After erythrocyte lysis and successive filtration through 250 and 40 µm sieves, SVF cells were resuspended in PBS containing 1% BSA and aliquots of 10^6^ cells were put into 96-well microtiter plates.

#### Fluorescence Activated Cell Sorting (FACS) on blood and SVF cells

Cells were pre-incubated (20 minutes on ice) with purified 2.4G2 monoclonal antibody in order to block Fc receptors (1∶50, BD Bioscience, San Diego, CA, USA). Cells were then incubated (45 minutes on ice) with primary antibodies (listed in Table S1) before being resuspended in PBS containing 0.5% BSA. Acquisition was made on 5×10^5^ cells using a FACS LSR Fortessa (BD Biosciences). Data were analyzed using the FlowJo 765 (Tree Star Inc.; Ashland, OR, USA).

#### Measurement of cytokine levels in adipose tissue explants and blood

For quantification of cytokines secreted by the visceral adipose tissue, tissue explants were cultured for 24 hours in DMEM without serum and supplemented with 1% of penicillin and streptomycin (50 U/ml, GIBCO, Life Technologies). Supernatants were harvested to quantify inflammatory cytokines (IL-6, MCP-1, TNFα and IL-10) using specific ELISA kits (R&D Systems). Blood IL-6, TNFα, MCP-1 and IL-10 levels were quantified using specific ELISA kits (R&D Systems).

### Gene Expression Analysis

#### RNA Extraction and Quality Assessment

Total RNA from liver, white and brown adipose tissue (WAT and BAT, respectively) was extracted using the Qiagen RNeasy Lipid Tissue kit (Qiagen, Germantown, MD, USA). RNAs were quantified and assessed for purity using a NanoDrop Spectrophotometer (Thermo Scientific, Wilmington, DE, USA). Integrity was verified with a BioAnalyser 2100 (Agilent, Palo Alto, CA, USA).

#### Microarray and Clustering Analysis

RNAs (140 ng) from pooled samples (n = 5/group) was amplified and cRNA was labeled with biotin using the TargetAmp Biotin-aRNA Labeling Kit (Illumina, Epicentre Biotechnologies, Madison, WI, USA). Biotin-labeled cRNA (1,500 ng) was hybridized to the Sentrix BeadChip Array for Gene Expression Mouse WG-6 V2 (Illumina) and incubated at 58°C for 16–20 hours in a hybridization oven (Illumina) with rocker speed at 5.

Beadchips were washed and stained according to manufacturer's protocol. Arrays were scanned by chip scanner Bead Array (Illumina), and images analyzed by Genome Studio (Illumina). Data were exported and processed using Genespring GX 11.5.1 (Agilent, Santa Clara, CA, USA). Data from HFD-Ve and HFD-Rapa mice were compared to data obtained from regular chow-fed mice. Genes that were differentially regulated in the experimental groups were identified using the *p*-value cut-off of 0.05. These genes were further analyzed using the Ingenuity Pathway software (Ingenuity, Redwood City, CA, USA). The microarray dataset has been submitted to Gene Expression Omnibus with accession number GSE53980.

#### Quantitative PCR

RNAs (400 ng) were reverse transcribed using the High Capacity cDNA Reverse Transcript Kit from Applied Biosystems (Foster City, CA, USA). Real-time (RT) quantitative PCR was performed on the ABI-7900HT Fast RT PCR system using SYBR green chemistry (Applied Biosystems). Primer sequences are available upon request. EeF2 was used as an internal control to normalize gene expression using the 2^−ΔCt^ method [40].

### Adipose Tissue and Liver Histological Analysis

#### Histology and Morphometric Analysis

Liver, visceral white (VWAT) and BAT samples were overnight fixed in 4% paraformaldehyde before embedding in paraffin. Multiple sections were obtained and stained with hematoxylin and eosin. For the morphometric analysis, at least 10 fields (representing approximately 100 adipocytes) per slide were analyzed. Images were acquired using an optical microscope (Axioplan 2 Imaging, Zeiss, Göttingen, Germany) and analyzed using the Image J software.

#### Immunohistochemical staining

VWAT tissue sections (5 mice per group) were stained with mAb anti-F4/80 (1∶500, eBioscience, San Diego, CA, USA). Staining was visualized using the Image J software. The specificity of the staining was verified by replacing each primary antibody by nonspecific IgG (Rat IgG1κ isotype Control (eBRG1) eBioscience).

### Isolation and Characterization of Myeloid-Derived Suppressor Cells (MDSCs) from Adipose Tissue and Liver

MDSCs were isolated and characterized from single-cell suspensions prepared from VWAT and liver of rapamycin- or Ve-treated HFD-fed mice using MACS technology. Single-cell preparations were washed twice with cold MACS buffer (1% BSA in PBS with 2 mmol/l EDTA). Cells (1×10^8^) were then resuspended in 350 µl MACS buffer and incubated with 100 µl biotinylated anti-Gr-1 Abs (Myeloid-Derived Suppressor Cells Isolation Kit, mouse from Miltenyi Biotec, Auburn, CA, USA) for 20 min at 4°C. Gr-1-labeled cells were then incubated at 4°C with 100 µl streptavidin microbeads (Miltenyi Biotec) for 15 min. Cells were washed, resuspended in 500 µl MACS buffer, and separated from unlabeled cells over a MACS column, according to the manufacturer's instructions (Miltenyi Biotec). The purity of cell preparations analyzed by flow cytometry was >90%.

### Protein Expression Analysis

Frozen WAT, liver and muscle samples were homogenized in RIPA lysis buffer (50 mmol/l Tris-HCL pH7.4, 1% NP-40, 0.5% Na deoxycholate, 0.1% SDS, 150 mmol/l NaCl, 2 mmol/l EDTA and 50 mmol/l NaF) supplemented wih 1× of protease inhibitors (cOmplete Protease Inhibitor Cocktail, Roche). After centrifugation of the lysates, protein concentration was estimated in the supernatant using a Bio-Rad Protein Assay kit (Bio-Rad Laboratories, Munich, Germany). Total proteins (40 µg) were separated on 10% SDS-polyacrylamide gel (Nu-PAGE, Invitrogen, Carlsbad, CA, USA) and then transferred to Immobilon-P Polyvinylidene fluoride membranes (Merck Millipore, Billerica, MA, USA). Membranes were blocked with 5% BSA in PBS 1× Tween 0.1% for one hour before being probed overnight at 4°C with specific primary antibodies (listed in Table S2). After washing, Ab binding was revealed by incubation with horseradish peroxidase (HRP)-conjugated secondary antibodies (2 hours incubation at room temperature, anti-Rabbit IgG HRP-linked antibody, 1∶5000, #7074, Cell Signaling) and ECL (ECL Plus Western Blotting Detection System, Amersham, Buckinghamshire, UK). Signals were quantified using the ImageJ software.

### Ethics Statement

Mice were maintained in a temperature-controlled (20±2°C) facility room with a strict daily cycle of 12 hours light and darkness and were given free access to food and water, unless stated.

Animals were housed in specific pathogen-free environment in Lille Pasteur Institute's animal facilities. Housing and experimentations were carried out according to the “Principles of laboratory animal care” (NIH publication n° 85-23, revised 1985; http://grants1.nih.gov/grants/olaw/references/phspol.htm) as well as to the French and European guidelines of laboratory animal care (European Communities Council Directive of 1986, 86/609/EEC) and approved by the Departmental Direction of Veterinary Services (Prefecture of Lille, France; IW's authorization number: 59-350152). Additionally, the present project has been submitted to, and approved by, the local Institutional Animal Care and Use Committee (CEEA 75) and received the authorization number AF 04/2010.

### Statistics

Data are presented as means ± SEM except when mentioned. The statistical significance of comparison between the different experimental groups was determined using the non-parametric Mann-Whitney U test. *p*-values less than 0.05 were considered statistically significant.

## Results

### Rapamycin reduced body weight gain and increased thermogenesis

**Figure 1:**
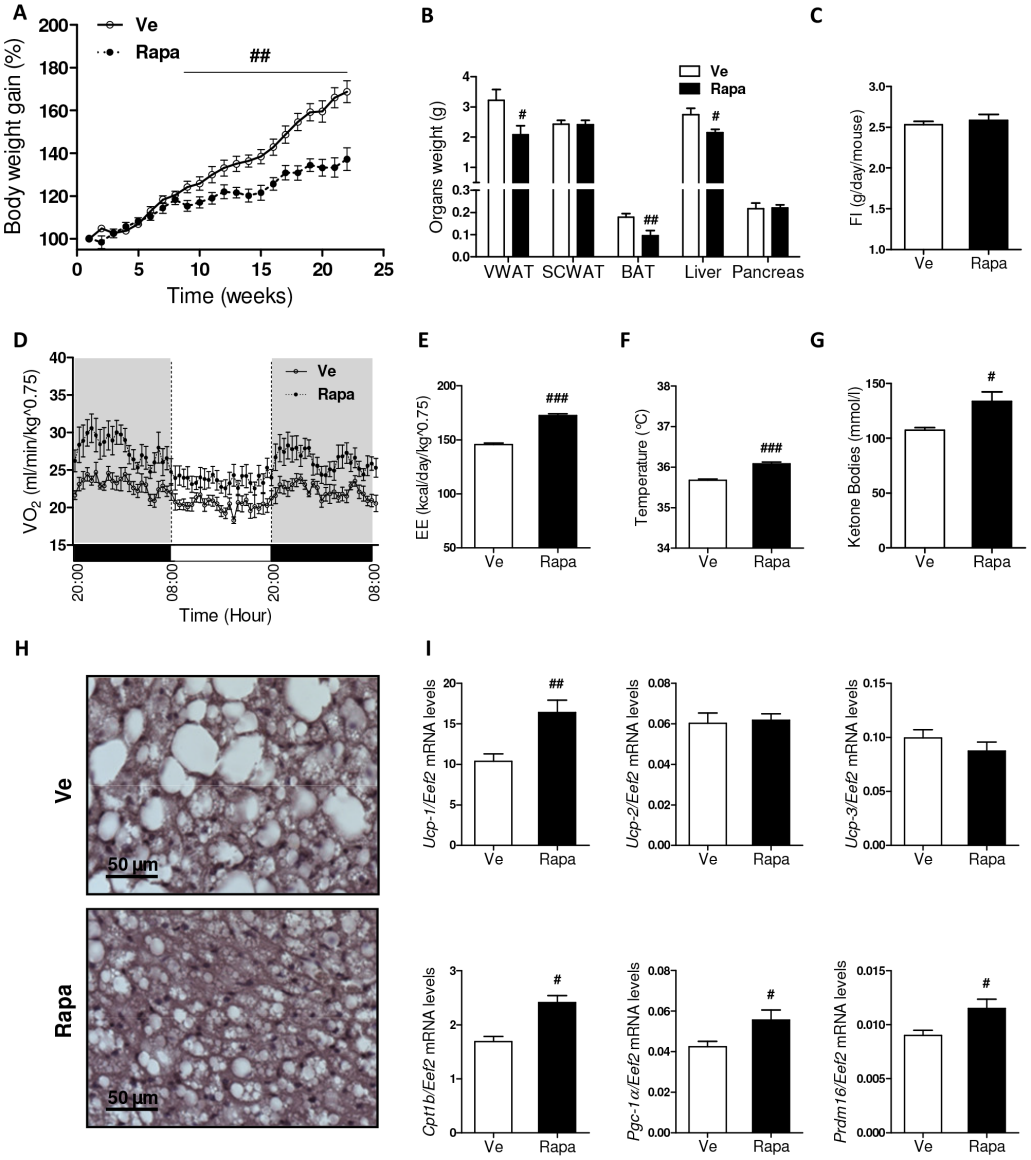
Body weight gain, feeding behavior and thermogenesis in rapamycin-treated mice. (**A**) Time course of body weight gain (%) measured over rapamycin treatment. Mice were fed on HFD for 6 weeks before receiving rapamycin (Rapa: •) or vehicle (Ve: ○) once a week for 22 weeks. (**B**) Masses (g) of visceral (perigonadal) white adipose tissue (VWAT), subcutaneous white adipose tissue (SCWAT), interscapular brown adipose tissue (BAT), liver and pancreas at 22 weeks (Rapa: ▪, Ve: □). (**C**) Cumulative food intake (g/day/mouse) (Rapa: ▪, Ve: □). (**D**) Oxygen consumption (Vo_2_) (ml/min/kg∧0.75) measured by indirect calorimetry over a 36-hour monitoring period (Rapa: •, Ve: ○). (**E**) Energy expenditure (kcal/day/Kg∧0.75) measured using indirect calorimetry over a 36-hour monitoring period (Rapa: ▪, Ve: □). (**F**) Core body temperature (°C) (Rapa: ▪, Ve: □). (**G**) Serum total ketone bodies (mmol/l) in 12-hours fasted mice (Rapa: ▪, Ve: □). (**H**) Representative sections of H&E-stained BAT of Ve- or Rapa-treated mice. Scale bars represent 50 µm. (**I**) Real-time quantitative PCR (RT-qPCR) analysis of BAT, after 22 injections (Rapa: ▪, Ve: □): Expression levels of *Ucp-1*, *Ucp-2*, *Ucp-3*, *Cpt1b*, *Pgc-1α* and *Prdm16* (normalized to *Eef2* expression). (**A–I**) Data are expressed as mean ± S.E.M. of 8 to 10 mice per group. ^#^ *p<*0.05, ^##^ *p<*0.01, ^###^ *p*<0.001.

After a 5-week period of high-fat diet feeding (HFD; 60% fat), mice were injected weekly (2 mg/kg/week) for 22 weeks with rapamycin (Rapa) or vehicle (Ve). Figure 1A shows that HFD-fed mice injected with rapamycin gained less weight than Ve mice. Consistent with body weight data, Rapa mice showed a significant reduction of visceral (perigonadal) white adipose tissue (VWAT) mass, compared to controls (Figure 1B). The lower leptin blood level in rapamycin-treated mice was concordant with reduced fat mass (Figure S1A).

Despite reducing body weight gain, rapamycin treatment did not affect food intake (Figure 1C), suggesting that increased energy expenditure might be the underlying cause of resistance to HFD in rapamycin-treated mice. To explore this, we housed mice from each group in metabolic cages in order to measure oxygen consumption (Vo_2_), carbon dioxide production (Vco_2_), and locomotor activity. We observed no consistent differences neither in respiratory quotient (RQ; an indirect indicator of lipid *vs.* carbohydrate utilization) nor in locomotor activity (Figures S1B and S1C). In contrast, Rapa mice consumed more oxygen than controls (Figure 1D) and showed higher energy expenditure (Figure 1E). Consistently, basal core body temperature and serum ketone bodies levels were higher in Rapa mice when compared to controls (Figures 1F and 1G). Strikingly, mass of the brown adipose tissue (BAT; the major site of thermoregulation) was lower in Rapa mice (Figure 1B) and its histological analysis revealed less lipid accumulation (Figure 1H), suggesting that rapamycin treatment has increased thermogenesis. This was comforted by significant increased expression of genes involved in BAT function, namely uncoupling protein-1 (*Ucp-1*; a mitochondrial protein specifically expressed in brown adipocytes that is involved in cold-induced nonshivering thermogenesis as well as diet-induced thermogenesis), carnitine palmitoyltransferase 1B (*Cpt1b*; a mitochondrial enzyme involved in fatty acid β-oxidation), peroxisome proliferator-activated receptor gamma coactivator 1-alpha (*Pgc-1α*; a factor that controls several aspects of mitochondrial biogenesis and is essential in BAT thermogenesis and PR domain containing 16 (*Prdm16*; a zinc-finger protein highly enriched in brown adipocytes that is crucial in the control of brown fat determination), whereas *Ucp-2* and *Ucp-3* expression levels were comparable in both groups (Figure 1I).

### Rapamycin increased macrophage recruitment in the adipose tissue

**Figure 2:**
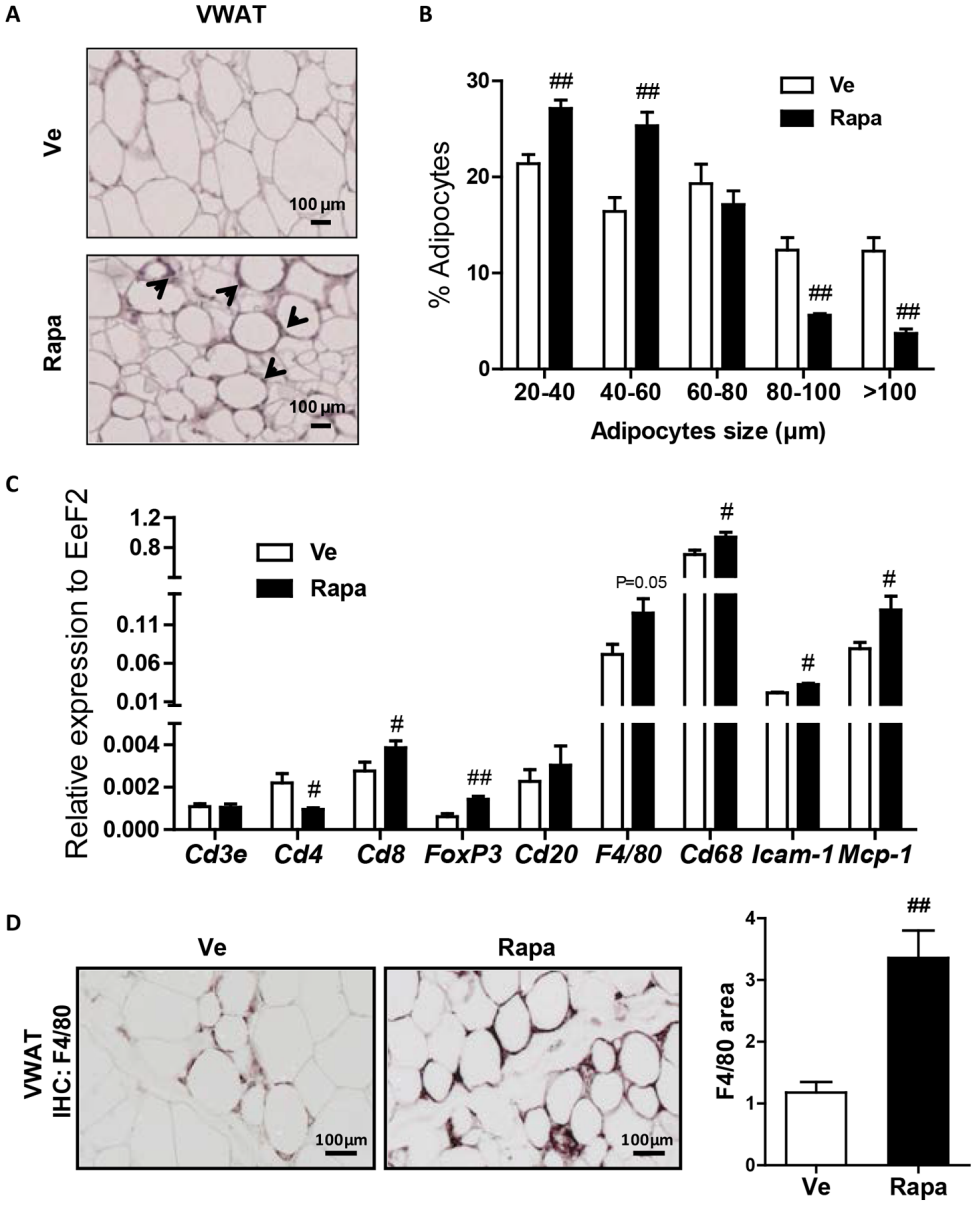
Rapamycin impact on VWAT. (**A**) Representative sections of H&E-stained VWAT of Ve- or Rapa-treated mice. Scale bars represent 100 µm. Black arrows target to infiltrating cells. (**B**) Adipocyte diameter distribution of the VWAT of 5 Ve-treated and 5 Rapa-treated HFD-fed mice (Rapa: ▪, Ve: □). (**C**) RT-qPCR analysis of VWAT, after 22 injections (Rapa: ▪, Ve: □): Expression levels of T-cell (*Cd4*, *Cd8*, *FoxP3*), B-cell (*Cd20*) and macrophage (*Cd68*, *F4/80*) specific markers and of leukocyte migratory factors (*Icam-1*, *Mcp-1*) specific markers (normalized to *Eef2* expression). Data are expressed as mean ± S.E.M. of 8 to 10 mice per group. **^#^** *p*<0.05, ^##^ *p*<0.01. (**D**) Representative sections of the VWAT from Ve- or Rapa-treated mice immunostained with F4/80 Ab (brown color). Scale bars represent 100 µm (left). Quantification of F4/80 positive signal on VWAT sections by Image J (Rapa: ▪, Ve: □) (right). Data are expressed as mean ± S.E.M. of 5 mice per group. ^##^ *p*<0.01.

Histological examination of VWAT sections showed a greater cell-size heterogeneity (Figure 2A) confirmed by morphometric analysis which showed a significant decrease in the percentage of large adipocytes (>80 µm) in the tissue of Rapa mice (Figure 2B). Strikingly, Rapa-treated mice VWAT was markedly infiltrated with cells, mostly located around the smallest adipocytes (Figure 2A).

To identify the nature of the cells infiltrating the VWAT of rapamycin-treated mice, we performed real-time quantitative PCR to compare the expression levels of genes specific for several immune cell-types known to invade the expanding adipose tissue (Figure 2C). While expression of *Cd3ε* (T lymphocytes) and *Cd20* (B lymphocytes) transcripts did not discriminate between rapamycin-treated and control mice, expression level of *Cd8* (cytotoxic T cells) transcript was significantly higher in VWAT from rapamycin-treated mice. In parallel, rapamycin significantly decreased the expression level of *Cd4* (T helper cells) while it increased that of *FoxP3* (Tregs), *Cd68* and *F4/80* (monocytes/macrophages) markers. The higher expression of the *Icam-1* and *Mcp-1*, two factors involved in leukocyte migration to inflamed tissues, indicated that rapamycin impacts on immune cell recruitment to VWAT [41], [42].

Immunohistochemistry analysis on VWAT sections revealed increased macrophage recruitment (Figure 2D). F4/80-positive cells (visualized by brown color) were organized as crown-like structures and the surface occupied by stained cells was 3-fold increased upon rapamycin treatment (*p*<0.01) (Figure 2D).

### Rapamycin impact on inflammation differs between tissues

**Figure 3:**
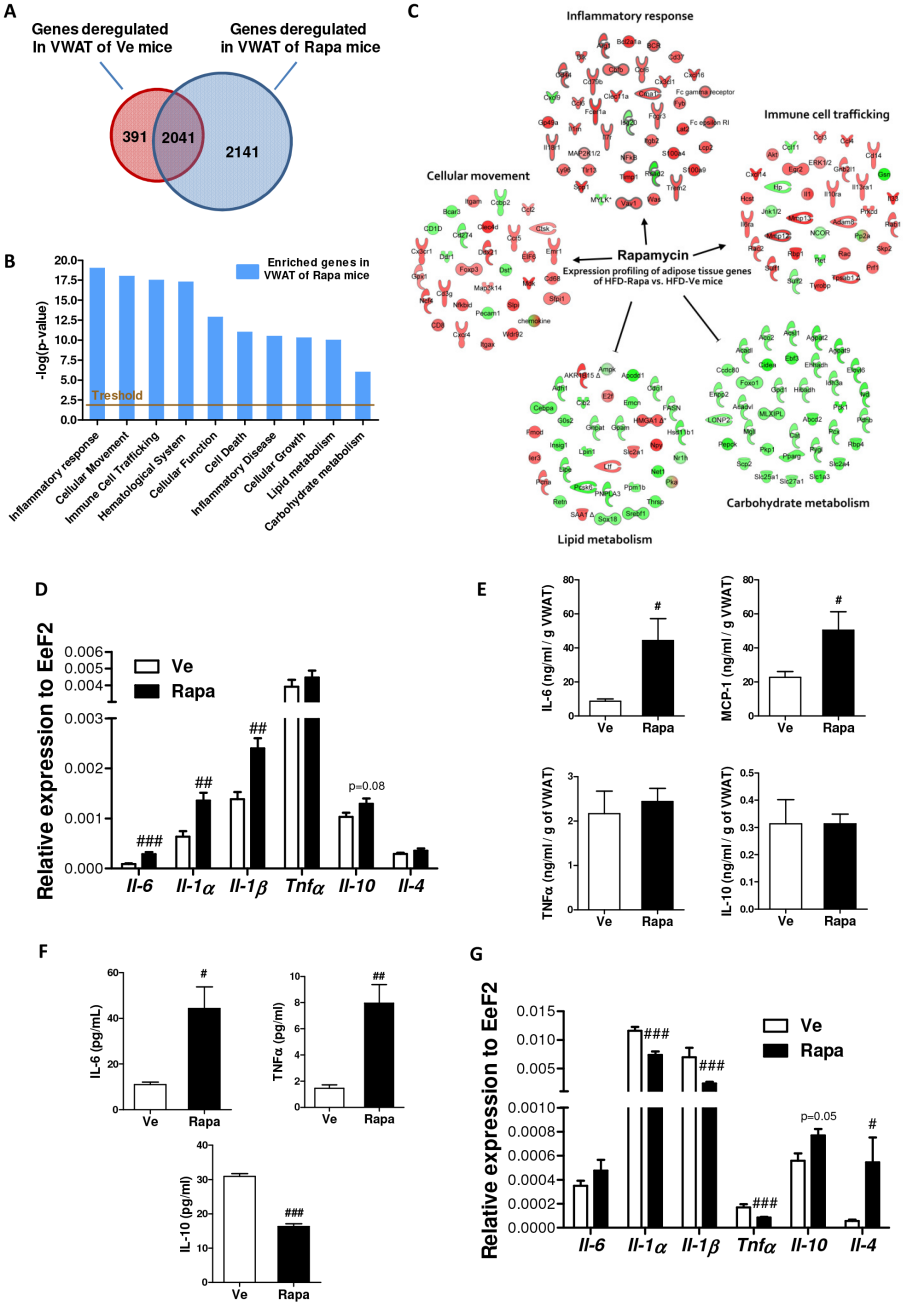
Tissue-specific effects of rapamycin on inflammation. (**A**) Venn diagrams of microarray data representing the number of genes deregulated in the VWAT from Ve- or Rapa-treated mice (respectively; red and blue circle). Genes deregulated at least by 1.5-fold at *p*<0.01 were considered for pathway analysis. (**B**) IPA analysis: Functional enrichment analysis showing the top 10 biological functions significantly deregulated in the VWAT of Rapa-treated mice compared to controls. (**C**) Expression profiling of genes implicated in the top deregulated pathways: downregulated genes (green) *versus* upregulated genes (red). (**D**) RT-qPCR analysis of VWAT, after 22 injections (Rapa: ▪, Ve: □): Expression levels of inflammatory cytokines (*Il-1α*, *Il-1β*, *Il-6* and *Tnfα*) and anti-inflammatory cytokines (*Il-4* and *Il-10*) (normalized to *Eef2* expression) (n = 8 to 10 mice per group). (**E**) IL-6, MCP-1, TNF-α and IL-10 levels in supernatants of VWAT explants (Rapa: ▪, Ve: □). ELISA values were normalized to the weight of VWAT explants and are expressed in ng/ml/g VWAT (n = 5 mice per group). (**F**) IL-6, TNF-α and IL-10 levels in blood (Rapa: ▪, Ve: □). ELISA values are in pg/ml (n = 8 to 10 mice per group). (**G**) RT-qPCR analysis of liver, after 22 injections (Rapa: ▪, Ve: □): Expression levels of inflammatory cytokines (*Il-1α*, *Il-1β*, *Il-6* and *Tnfα*) and anti-inflammatory cytokines (*Il-4* and *Il-10*) (normalized to *Eef2* expression) (n = 8 to 10 mice per group). (**D–G**) Data are expressed as mean ± S.E.M. **^#^** *p*<0.05, ^##^ *p*<0.01, **^###^** *p*<0.001.

To assess the inflammatory status of the adipose tissue, we first performed microarray gene expression analysis in VWAT. Compared to gene expression in the VWAT of mice fed with regular chow, 2,432 genes were deregulated by HFD (Ve group) and this number was increased by ∼1.5-fold (*i.e.* 4,182 genes) upon rapamycin injection (Rapa group), as represented by Figure 3A. Genes specifically deregulated in the VWAT of Rapa mice (2,141 genes; *p*<0.01) were included in Ingenuity Pathway Analysis (IPA) for pathway identification. As shown by Figure 3B, the most significant deregulated pathways are related to inflammatory response, cellular movement and immune cell trafficking. Furthermore, pathways related to inflammatory response and immune cell trafficking were predicted to be upregulated in the VWAT of rapamycin-treated mice (Figure 3C and Table S3). In contrast, pathways related to lipid or carbohydrate metabolism were predicted to be under-expressed (Figure 3C).

Then, we assessed the expression levels of pro-inflammatory cytokines (*e.g. Il-1α*, *Il-1β*, *Il-6* and *Tnfα*) and anti-inflammatory cytokines (*e.g. Il-4* and *Il-10*) transcripts in the VWAT. Figure 3D shows that rapamycin had no discernible effect on the expression of *Tnfα*, *Il-4* and *Il-10*, yet it markedly enhanced the expression of *Il-6* (3-fold increase), *Il-1α*and *Il-1β* (2-fold increase). The expression data were further supported by protein secretion analysis from VWAT explants which showed that adipose tissue samples from rapamycin-treated mice secreted more IL-6 and MCP-1 than control explants whereas the amount of TNFα and IL-10 was comparable (Figure 3E).

Adipose tissue inflammation was associated with systemic inflammation, as revealed by higher blood levels of IL-6 and TNFα and decreased level of IL-10 in Rapa mice, compared to controls (Figure 3F). In the liver however, rapamycin treatment significantly decreased the expression of *Il-1α* (1.5-fold), *Il-1β* (3-fold) and *Tnfα* (2-fold) transcripts whilst that of *Il-10* tended to increased, almost reaching statistical significance (*p* = 0.053) and that of *Il-4* increased by 9-fold (Figure 3G).

### Rapamycin increased insulin sensitivity

We then assessed whether the increased inflammatory profile of rapamycin-treated mice impacts on lipid and glucose homeostasis. Rapamycin slightly decreased fasting triglyceride levels in the blood of rapamycin-treated mice but had no effect on glycerol and non-esterified fatty acid (NEFA) blood levels (Table S4 and Materials and Methods S1).

**Figure 4:**
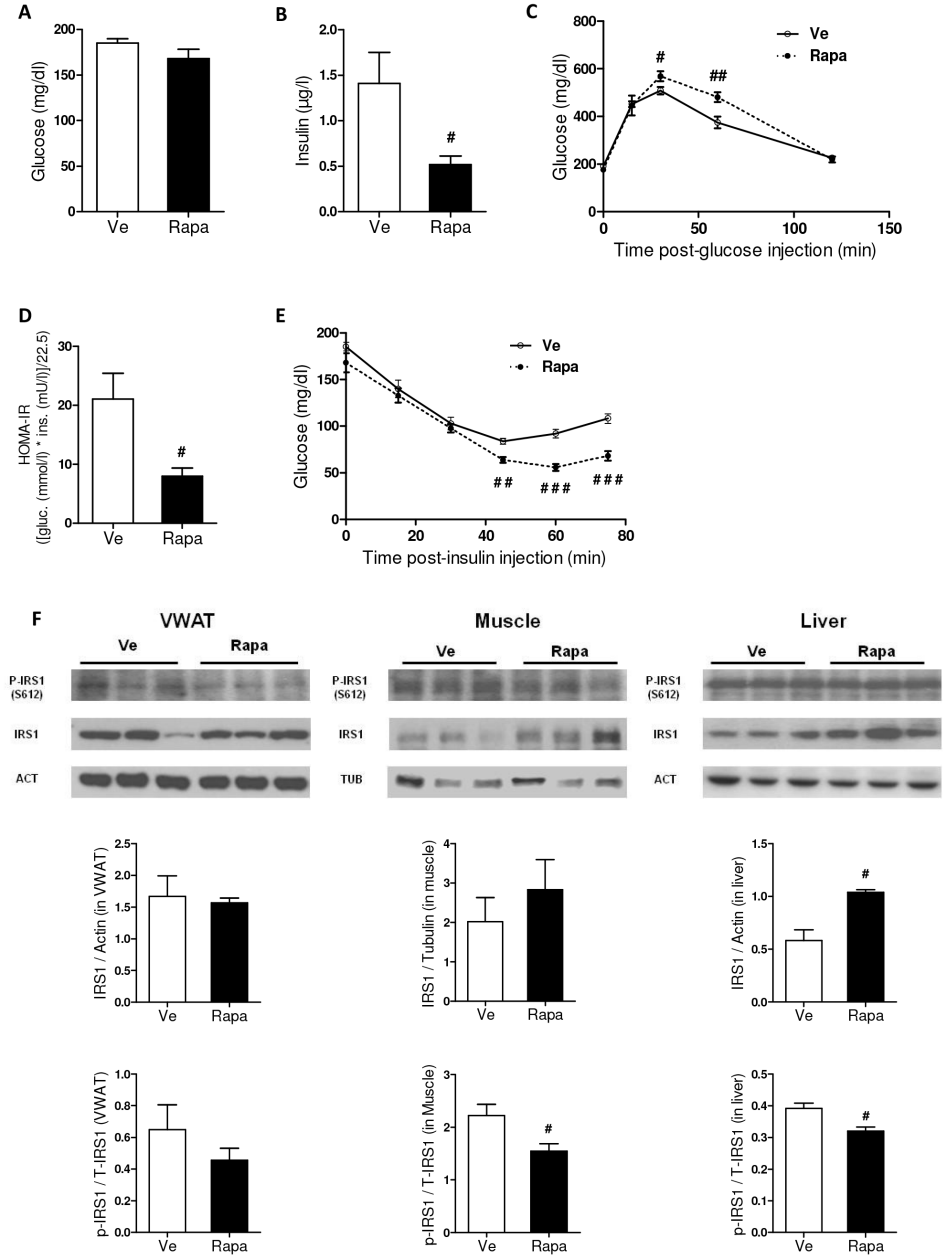
Effect of rapamycin on glucose homeostasis. (**A**) Fasted glucose levels (mg/dl) at week-17 post-injection (Rapa: ▪, Ve: □). (**B**) Fasted insulin levels (µg/l) at week-17 post-injection (Rapa: ▪, Ve: □). (**C**) Glucose tolerance test (GTT) at week-16 post-injection (Rapa: •, Ve: ○). Blood glucose levels (mg/dl) were measured in 6 hours-fasted mice (T0) and at the indicated times following intra-peritoneal (i.p.) injection of glucose. (**D**) Homeostatic model for assessment of insulin resistance (HOMA-IR) (Rapa: ▪, Ve: □). HOMA-IR = (17^th^ week post-injection-fasted Serum Insulin x 17^th^ week post-injection-fasted Serum Glucose)/22.5. (**E**) Insulin tolerance test (ITT) at week-18 post-injection (Rapa: •, Ve: ○). Mice were fasted for 6 hours before being i.p. injected with insulin. Blood glucose levels (mg/dl) were measured from T = 0 to T = 75 minutes after insulin administration. (**F**) Western-blot analysis of total and phosphorylated IRS-1 in VWAT, muscle and liver. Each lane represents an individual mouse (5 mice per group were analyzed, 3 representative mice per group are figured). β-actin was used as internal control for VWAT and liver samples and tubulin serves as control for muscle. Quantification of the signals was done using Image J (Rapa: ▪, Ve: □). Data are expressed as mean ± S.E.M. of 3 mice per group. ^#^ *p*<0.05. (**A–E**) Data are expressed as mean ± S.E.M. of 8 to 10 mice per group. **^#^** *p*<0.05, **^##^** *p*<0.01, ^###^ *p<*0.001.

We next examined the effects of rapamycin upon fasting glucose and glucose tolerance after glucose administration in mice. As shown in Figure 4, rapamycin had no effect on basal fasting glucose yet it decreased fasting insulin (Figures 4A and 4B). Although rapamycin treatment did not improve glucose intolerance (Figure 4C), homeostatic model for assessment of insulin resistance (HOMA-IR) values derived from both insulin and glucose levels were significantly reduced (Figure 4D), indicating higher insulin sensitivity which was confirmed by an insulin tolerance test (Figure 4E). These findings indicate that upon rapamycin treatment, HFD-fed mice progressed from being insulin resistant to have improved insulin sensitivity, as demonstrated by the decreased S612 phosphorylation of IRS-1 (insulin receptor substrate-1) in adipose tissue, muscle and liver (Figure 4F). Indeed, decreased phosphorylation of ISR-1 at serine 612 will avoid IRS-1 degradation and, thus, maintain insulin sensitivity [43].

### Rapamycin impact on immune regulatory cells in blood, adipose tissue and liver

Despite elevated systemic and VWAT inflammation, HFD-fed rapamycin-treated mice developed less insulin resistance than HFD-Ve mice. Therefore, we investigated whether rapamycin might have promoted the accumulation of immunosuppressive, anti-inflammatory regulatory cells in blood, adipose tissue and liver.

**Figure 5:**
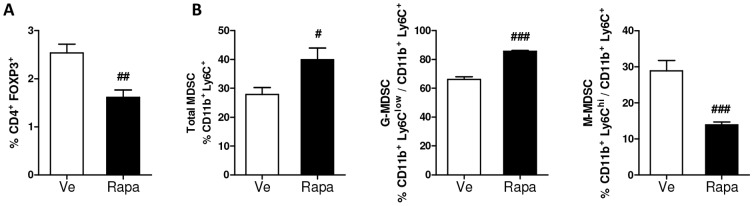
Effect of rapamycin on T cell and myeloid-derived suppressor cell (MDSCs) subsets in blood (22 weeks post-injection). (**A**) CD4^+^ FoxP3^+^ regulatory T-cells (Tregs) were analyzed in the blood by flow cytometry after gating on total live cells (Rapa: ▪, Ve: □). Results are expressed as a percentage of live cells. (**B**) Total MDSCs (Ly6C^+^ CD11b^+^), granulocytic MDSCs (G-MDSCs; CD11b^+^ Ly6G^+^Ly6C^low^) and monocytic MDSCs (M-MDSCs; CD11b^+^ Ly6G^−^ Ly6C^hi^) were analyzed in the blood by flow cytometry after gating on total live cells (Rapa: ▪, Ve: □). Results are expressed as a percentage of live cells. (**A–B**) Data are expressed as mean ± S.E.M. of 8 mice per group. ^#^ *p<*0.05, ^##^ *p<*0.01.

In blood, the percentage of CD4^+^ FoxP3^+^ regulatory T cells (Tregs) was significantly reduced in the group of rapamycin-treated animals (Figure 5A). In opposite, the percentage of total MDSCs (identified by CD11b and Ly6C co-staining) was enhanced. Furthermore, within MDSCs the proportion of granulocytic MDSCs (G-MDSCs; identified as CD11b^+^ Ly6G^+^ Ly6C^low^ cells) increased while that of monocytic MDSCs (M-MDSCs; identified as CD11b^+^ Ly6G^−^ Ly6C^hi^ cells) decreased (Figure 5B). Of note, no significant changes were noticed in the percentage of CD4^+^, CD8^+^ T -cells or B -cells between groups (Figures S2A, S2B and S2C).

**Figure 6:**
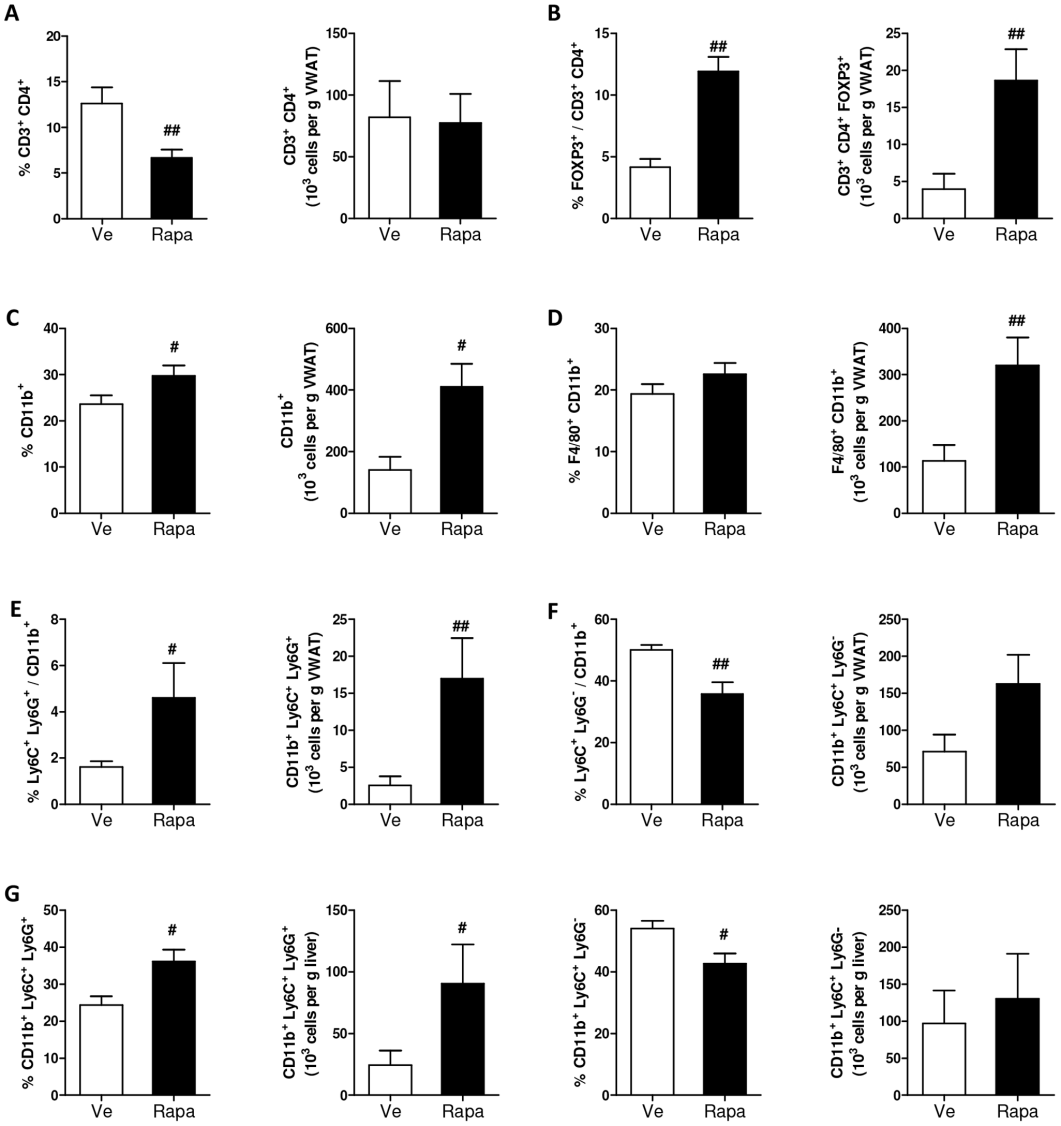
Effect of rapamycin on T cell and myeloid-derived suppressor cell (MDSCs) subsets in adipose tissue stromal vascular fraction (SVF) (22 weeks post-injection). (**A**) Adipose tissue CD3^+^ CD4^+^ T -cells were analyzed by flow cytometry and expressed as a percentage of live cells (after exclusion from the analysis of B220^+^, CD19^+^ and CD11c^+^ cells) (left panel) or as a number (×10^3^)/VWAT mass (g) (right panel) (Rapa: ▪, Ve: □). (**B**) Adipose tissue CD4^+^ FoxP3^+^ regulatory T cells (Tregs) were analyzed by flow cytometry and expressed as a percentage of CD3^+^ CD4^+^ cells (left panel) or as a number (×10^3^)/VWAT mass (g) (right panel) (Rapa: ▪, Ve: □). (**C**) Adipose tissue CD11b^+^ cells were analyzed by flow cytometry. Results are expressed as a percentage of live cells (after exclusion of T and B lymphocytes from the analysis) (left panel) or as a number (×10^3^)/VWAT mass (g) (right panel) (Rapa: ▪, Ve: □). (**D**) Adipose tissue F4/80-expressing macrophages were analyzed by flow cytometry. Results are expressed as a percentage of CD11b^+^ cells (left panel) or as a number (×10^3^)/VWAT mass (g) (right panel) (Rapa: ▪, Ve: □). (**E**) Adipose tissue granulocytic MDSCs (G-MDSCs; CD11b^+^ Ly6G^+^Ly6C^low^) were analyzed by flow cytometry. Results are expressed as a percentage of CD11b^+^ or as a number (×10^3^)/VWAT mass (g) (right panel) (Rapa: ▪, Ve: □). (**F**) Adipose tissue monocytic MDSCs (M-MDSCs; CD11b^+^ Ly6G^−^ Ly6C^hi^) were analyzed by flow cytometry. Results are expressed as a percentage of CD11b^+^ or as a number (×10^3^)/VWAT mass (g) (right panel) (Rapa: ▪, Ve: □). (**G**) M-MDSC and G-MDSC populations in the liver were analyzed by flow cytometry. Results are expressed as a percentage of CD11b^+^ (Rapa: ▪, Ve: □). (**A–G**) Data are expressed as mean ± S.E.M. of 7 to 9 mice per group. ^#^ *p<*0.05, ^##^ *p<*0.01, ^###^ *p<*0.001.

In the adipose stromal vascular fraction, rapamycin significantly decreased by 2-fold the percentage of CD4^+^ T -cells yet absolute numbers normalized to fat pad weight were comparable between Rapa and Ve mice (Figure 6A). However, in contrast to blood, in adipose tissue rapamycin increased by 2-fold the percentage of CD4^+^ FoxP3^+^ regulatory T cells. This enhancing effect of rapamycin on Tregs was even more pronounced after normalization to fat pad mass (Figure 6B). Regarding cells of myeloid origin, we found that the percentage of CD11b^+^ cells was slightly higher, this increase being more pronounced after normalization to fat mass weight (Figure 6C). Among CD11b^+^ cells, the number of F4/80^+^ cells was significantly increased in rapamycin-treated mice when normalized to fat mass (Figure 6D), supporting our immunohistochemical observation (Figure 2D). Importantly, macrophages of rapamycin-treated mice expressed lower levels of CD11c, a marker of pro-inflammatory M1 macrophage subset (Figure S3).

When assessing for MDSCs we showed that, in adipose tissue, rapamycin increased by 2-fold the percentage of G-MDSCs (Figure 6E), as well as found in blood, whereas the percentage of M-MDSCs was decreased yet their absolute numbers remained unchanged (Figure 6F).

Importantly, the positive impact of rapamycin on G-MDSCs that was evidenced in blood and adipose tissue also hold true for the liver (Figure 6G).

**Figure 7:**
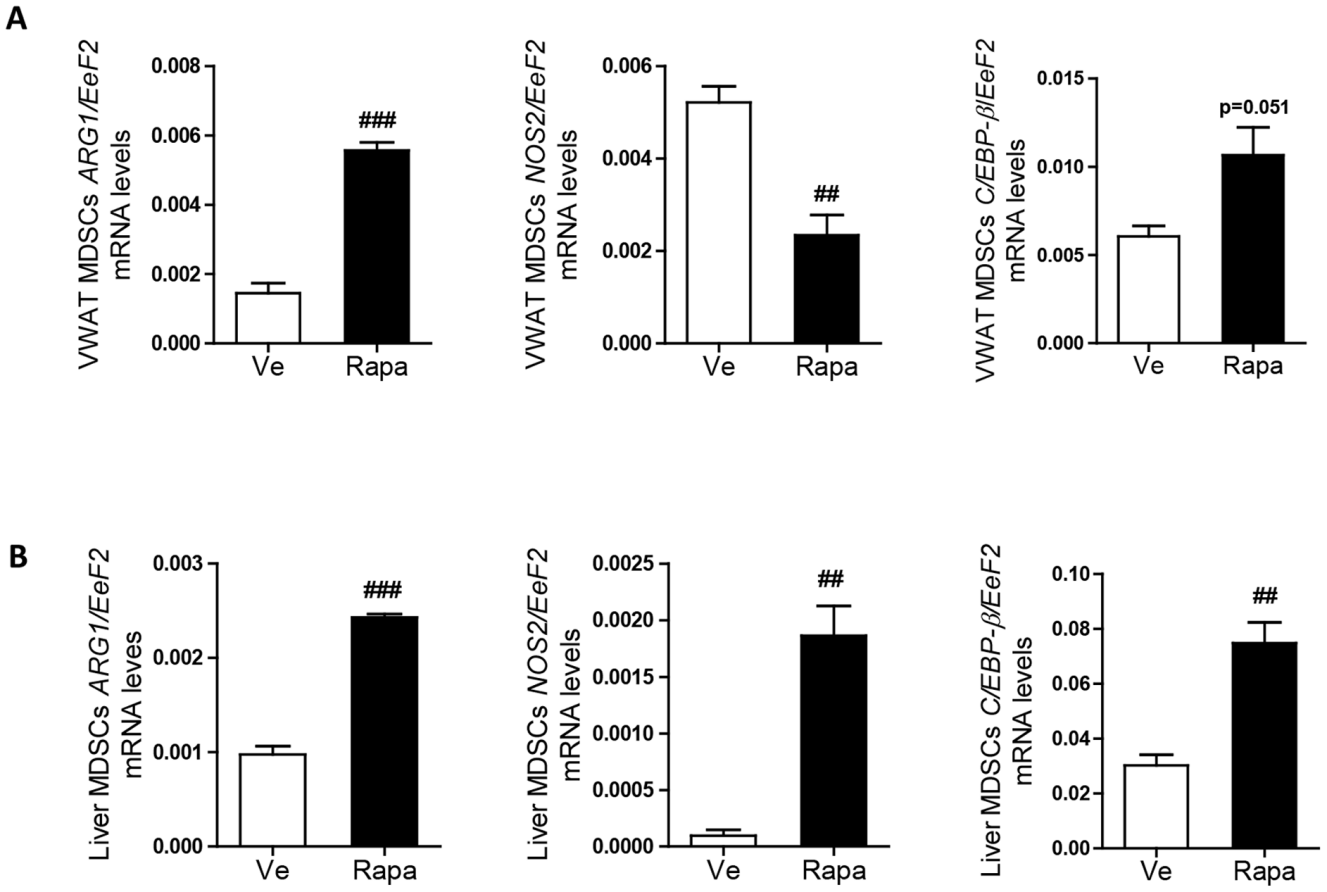
Effect of rapamycin on the expression of MDSCs specific genes by MDSCs purified from adipose tissue and liver (22 weeks post-injection). (**A**) RT-qPCR analysis of MDSCs purified from VWAT, after 22 injections (Rapa: ▪, Ve: □): Expression levels of *Arg1*, *Nos2* and *C/EBP-β* (normalized to *Eef2* expression) (n = 5 mice per group). (**B**) RT-qPCR analysis of MDSCs purified from the liver, after 22 injections (Rapa: ▪, Ve: □): Expression levels of *Arg1*, *Nos2* and *C/EBP-β* (normalized to *Eef2* expression) (n = 5 mice per group). (**A**–**B**) Data are expressed as mean ± S.E.M. of 5 mice per group. ^##^ *p<*0.01, ^###^ *p<*0.001.

Aiming to provide evidence regarding the functional immunoregulatory properties of MDSCs in adipose tissue and liver, we isolated these cells using the MACS technology. Analysis of the expression of genes specifically expressed by MDSCs showed increased expression of *Arg1* and *C/EBP-β* transcripts in the MDSCs purified from the VWAT and the liver of rapamycin-treated mice. In addition, while *Nos2* expression was decreased in VWAT, it was significantly increased in the liver (Figures 7A and B). These results confirmed that rapamycin favored immunoregulatory functions of MDSCs in adipose tissue and liver, thereby impacting their inflammatory and, consequently, insulin sensitive states.

### Effect of rapamycin on mTORC1 and mTORC2 activities in adipose tissue, muscle and liver

**Figure 8:**
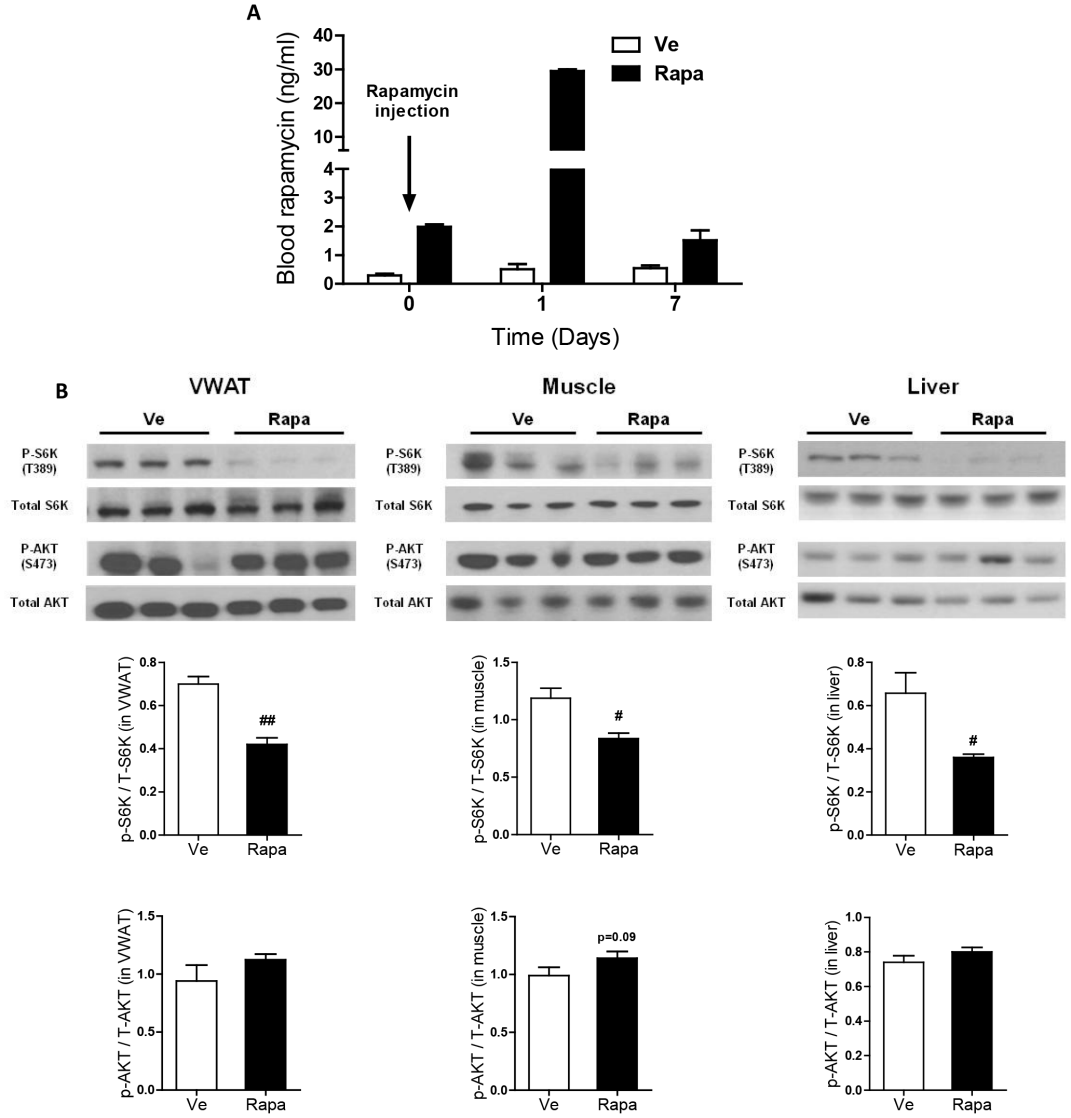
Effect of rapamycin on mTORC1 and mTORC2 activities. (**A**) Rapamycin blood levels (Rapa: ▪, Ve: □). Mice were bled before, and at days 1 and 7 post-injection. Results are expressed as mean ± S.E.M. of 2 mice per group. (**B**) Western blot analysis of S6K1 (total and T389-phosphorylated) and AKT (total and S437-phosphorylated) in VWAT, muscle and liver. Each lane represents an individual mouse (5 mice per group were analyzed, 3 representative mice per group are figured). Quantification of the signals was done using Image J (Rapa: ▪, Ve: □). Data are expressed as mean ± S.E.M. of 3 mice per group. ^#^ *p*<0.05.

To better define the respective actions of mTORC1 and mTORC2 on the observed effects, it was crucial to determine whether and how our protocol affected rapamycin levels and inhibited mTOR complexes. Quantification of rapamycin blood levels showed that between each injection, rapamycin returns to basal levels signifying that drug accumulation did not occur (Figure 8A and Materials and Methods S1). Moreover, a biochemical analysis of VWAT, muscle and liver showed that the rapamycin injection regimen decreased phosphorylation of S6K1 at T389 (direct down-stream target of mTORC1) thus indicating that mTORC1 is inhibited. However, rapamycin treatment did not affect AKT phosphorylation at S473 (a down-stream target of mTORC2), which may indicate that mTORC2 activity is conserved [44] (Figure 8B).

## Discussion

Rapamycin, an immunosuppressive drug that prevents organ transplant rejection [4], is a potent inhibitor of the mTOR pathway. Many studies have demonstrated the effect of rapamycin on metabolism in lean and obese rodents. In contrast, no study has yet addressed whether rapamycin metabolic effects are associated with its immunoregulatory properties. Here we showed beneficial metabolic effects of chronic rapamycin administration to HFD-fed mice that are associated with increased levels of Tregs and MDSCs in metabolic tissues. Furthermore, we suggest that these metabolic and immunological features are primarily due to rapamycin actions on mTORC1.

Compared to controls, rapamycin-treated mice were leaner with enhanced insulin sensitivity, increased oxygen consumption and ketogenesis. However, rapamycin-treated HFD-fed mice showed increased glucose intolerance due to lower basal insulin levels, decreased expression levels of GLUT4 in the adipose tissue and, in the liver, decreased lipogenesis and increased gluconeogenesis (Figures S1D and S4).

Inhibition of mTORC1 underlies most, yet not all, of these beneficial metabolic effects. Indeed, mTORC1 exerts a positive role in adipogenesis [1], [11] and we accordingly showed decreased expression of pro-adipogenic factors in the adipose tissue of rapamycin-treated animals (Figure S1D), in line with the reduced adipocyte size evidenced in the VWAT of Rapa mice (Figures 2A and 2B). Increased thermogenesis may also result from mTORC1 inhibition since adipose tissue conditional knockout of this complex results in lean mice with increased energy expenditure; phenocopying our rapamycin-treated animals [11]. Furthermore, the increased thermogenesis of rapamycin-treated mice likely resulted from enhanced BAT activity and not from higher muscle thermogenesis. Indeed, we showed altered BAT histology and gene expression pattern in rapamycin-treated mice and it has been reported that mTORC1 inhibition in muscle reduces mitochondrial biogenesis [45], [46].

Inhibition of mTORC1 partly explains the insulin sensitive state of rapamycin-treated mice, both through abrogating IRS-1 degradation and through affecting pancreatic β-cell insulin secretion [1]. However, mTORC2 activity is crucial to maintain insulin sensitivity, as revealed by the insulin resistant state of mice in which mTORC2 is inhibited [47]. Thereby, mTORC2 is likely not affected in rapamycin-treated mice, as indicated by the conserved serine phosphorylation of AKT (a down-stream target of mTORC2). Nevertheless, assessing additional downstream targets of mTORC1 and mTORC2 pathways (such as, respectively, 4EBP1 and PKCα [1]) would reinforce the demonstration that rapamycin treatment inhibited mTORC1 and not mTORC2.

Strikingly, despite improved insulin sensitivity, systemic and adipose tissue inflammation were increased in rapamycin-treated mice. In line with enhanced inflammation, we found reduced adiponectin blood levels in Rapa mice (Figure S1A). Indeed, inflammatory cytokines such as IL-6 or TNFα have been shown to decrease adiponectin secretion by adipose cells and this, despite reduced fat mass [48], [49]. Even if intriguing, the uncoupling of inflammation and insulin resistance has been already reported in several experimental settings [50]–[54]. For example, mice with elevated NF-κB activity in adipose tissue displayed increased WAT and systemic inflammation, increased energy expenditure and enhanced insulin sensitivity [55], as we observed in our model. It suggests that inflammation may have either negative (*e.g.* inhibition of insulin sensitivity [27], [29]–[31]) or positive (*e.g.* induction of energy expenditure (current study and [44]–[49]) activities in the regulation of metabolism. Our study indicates that rapamycin-induced increase in energy expenditure may antagonize insulin resistance partly by increasing the number of regulatory cells (*e.g.* Tregs and MDSCs) in metabolic tissues such as WAT and liver. Besides, we showed that inflammation was decreased in the liver – an insulin-sensitive tissue that plays the major role in whole body insulin sensitivity – with beneficial effect on hepatic insulin sensitivity, as supported by the decreased serine phosphorylation of IRS-1 (Figure 4F).

Interestingly, the beneficial metabolic effects of rapamycin were associated with immune changes, mostly affecting cells with immunoregulatory functions such as regulatory T cells and MDSCs. Indeed, rapamycin altered the immune cell composition of blood, adipose tissue and liver. The enhanced proportion of regulatory cells in the adipose tissue and liver of rapamycin-treated mice is also thought to participate to the beneficial effect of the drug on metabolic parameters. Obesity results in adipose-resident FoxP3^+^ Treg depletion [37] and gain-of-function experiments to expand their numbers improved insulin sensitivity [37], [56]. mTOR has recently emerged as a critical regulator of Tregs, acting both on their *de novo* generation and on their selective expansion [19], depending on the modulation of mTOR complexes [21], [22]. Furthermore, it has been reported that leptin plays a key role in the control of mTOR activity in Tregs [23], [57]. Our results showed that rapamycin treatment decreased leptin blood levels and promoted Tregs in the adipose tissue, the cells being recruited from the blood (Figure 5), suggesting that the leptin-mTOR axis may be involved in the enhanced generation of Tregs in rapamycin-treated animals.

Beside Tregs, MDSCs were lately reported to also provide a key check-and-balance platform to counter inflammation and insulin resistance in obesity. Indeed, down-regulation of MDSCs in obese animals leads to the deterioration of insulin sensitivity whereas elevation of these cells has the opposite effect [33]. Supporting this hypothesis, in rapamycin-treated mice we found an increased number of MDSCs in blood, liver and adipose tissue. The immunosuppressive functions of Tregs and MDSCs in metabolic tissues of rapamycin-treated mice remain to be fully determined. However, we showed an increased expression of genes involved in the immunoregulatory functions of MDSCs in cells enriched from the WAT and liver of rapamycin-treated animals (Figure 7). While the role of mTOR pathway in MDSCs is still unknown, rapamycin may have blunted Tregs, since mTORC1 was demonstrated to be crucial for their functionality *in vivo* [58]. Furthermore, in order to dissociate the effect of mTOR inhibition on insulin sensitivity from its impact on the recruitment of regulatory cells to metabolic tissues, selective depletions of either regulatory cell-types could be envisaged.

In conclusion, the present work assessed for the first time the metabolic and immune consequences of rapamycin administration to obese mice. It showed that rapamycin improved metabolic parameters partly through acting on regulatory immune cells; thereby revealing a novel aspect of the immunoregulatory properties of rapamycin. Several previous studies on the metabolic effect of chronic rapamycin injection were controversial depending on the animal model (rat *versus* mouse), animal strain, diet, and age as well as rapamycin dose, route and frequency of administration [17], [59], [60]. Here we show that our protocol, which can be qualified as “chronic intermittent”, has beneficial effects on energy homeostasis of mice as recently reported by Fang *et al*. who used another mouse strain in a context of standard diet, a condition in which mTOR is not over-activated as it is in obesity [16].

Even if the relative contribution of liver, muscle and adipose tissue remains to be established as well as rapamycin molecular mechanism of action, we propose however that the protective metabolic effect of rapamycin may partly rely on immune modifications induced by this immunoregulatory drug, such as the increase of regulatory cells like Tregs and MDSCs. Our work underscores the interest of targeting the mTOR pathway for clinical applications in humans to limit deleterious metabolic side effects, perhaps through the development of novel mTOR inhibitors that selectively inhibit mTORC1.

## Supporting Information

Figure S1 **Effect of rapamycin on adiponectin and leptin blood levels, on respiratory quotient and locomotor activity and expression of adipogenic factors.** (**A**) Adiponectin and leptin blood levels (Rapa: ▪, Ve: □). (**B**) Respiratory quotient (Rapa: ▪, Ve: □) and (**C**) locomotor activity, measured over a 36-hour monitoring period (16-week post-injection). (**C**) Real-time quantitative PCR (RT-qPCR) analysis of the VWAT of Ve- or Rapa-treated mice, after 22 injections (Rapa: ▪, Ve: □): Expression levels of *PPARγ*, *C/EBPa*, *SREBP-1c*, *FAS*, *leptin* and *GLUT4*. Data are expressed as mean ± S.E.M. of 8 to 10 mice per group. ^#^ *p*<0.05, ^##^ *p<*0.01, ^###^ *p*<0.001 ^####^ *p<*0.0001.
(PDF)

Figure S2 **Effect of rapamycin on blood lymphocyte T CD4^+^, CD8^+^ and B cells.** (**A**) CD3^+^ CD4^+^ T-cells, (**B**) CD3^+^ CD8^+^ T-cells, and (**C**) CD19^+^ B220^+^ B-cells were analyzed in the blood by flow cytometry (Rapa: ▪, Ve: □). Results are expressed as percentage of live cells. Data expressed as mean ± S.E.M. of 8 mice per group.
(PDF)

Figure S3 **Effect of rapamycin on CD11b^+^ F4/80^+^ CD11c^+^ subset in adipose tissue stromal vascular fraction (SVF) (22 weeks post-injection).** Adipose tissue CD11c-expressing macrophages were analyzed by flow cytometry. Results are expressed as percentage of CD11b^+^ F4/80^+^ cells (left panel) or as a cell number (×10^3^)/VWAT mass (g) (right panel) (Rapa: ▪, Ve: □).
(PDF)

Figure S4 **Liver histology and gluconeogenesis-related gene expression.** (**A**) Representative sections of H&E-stained liver (Scale bars represent 100 µm). (**B**) Real-time quantitative PCR (RT-qPCR) analysis of the liver, after 22 injections (Rapa: ▪, Ve: □): Expression levels of the gluconeogenesis-related genes *PEPCK*, *PGC-1α* and *G6PC*. Data are expressed as mean ± S.E.M. of 8 to 10 mice per group. ^#^ *p<*0.05.
(PDF)

Materials and Methods S1 **Glycerol, triglycerides, non-esterified fatty acids (NEFAs) and Sirolimus blood levels.** Glycerol, triglycerides, non-esterified fatty acids (NEFAs) and Sirolimus blood levels were quantified using, respectively, colorimetric and chemiluminescence assays.
(DOC)

Table S1 **Antibodies used for FACS.** Primary antibodies used for FACS analysis (clones, dilution, origin).
(PDF)

Table S2 **Antibodies used for western-blot.** Primary antibodies used for western blot analysis (dilution, origin).
(PDF)

Table S3 **Ingenuity Pathway Analysis (IPA) of deregulated genes in Rapamycin VWAT.** Top enriched pathways in top enriched biological functions: Immune cell trafficking and Inflammatory response (*p* values, predicted activation state, # molecules).
(PDF)

Table S4 **Blood lipid profiles in rapamycin-treated mice.** Triglycerides, glycerol and non-esterified fatty acids (NEFAs) blood levels (in mmol/l). Results are expressed as mean ± S.E.M. of 8 to 10 mice per group. ^#^ *p*<0.05.
(PDF)
