# Supplementary figures and images for: Beneficial Metabolic Effects of Rapamycin Are Associated with Enhanced Regulatory Cells in Diet-Induced Obese Mice
Source: PLoS One. 2014 Apr 7;9(4):e92684. doi: 10.1371/journal.pone.0092684 (PMC3977858; doi:10.1371/journal.pone.0092684)

**A**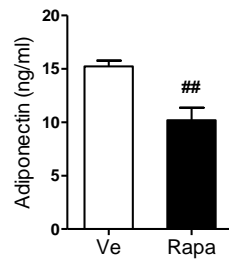**B**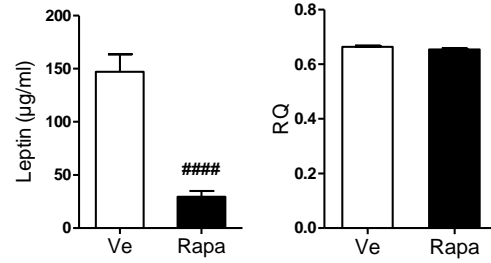**C**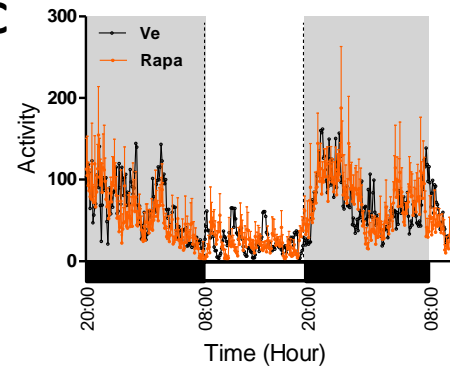**D**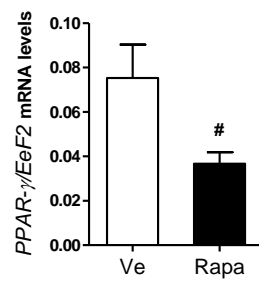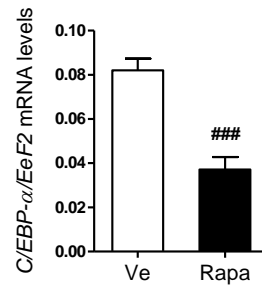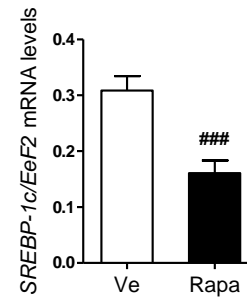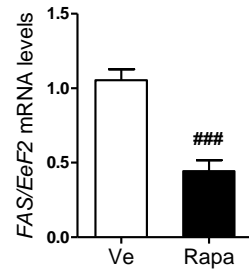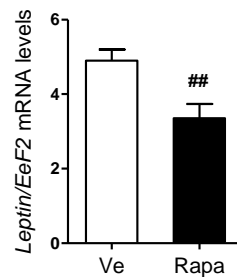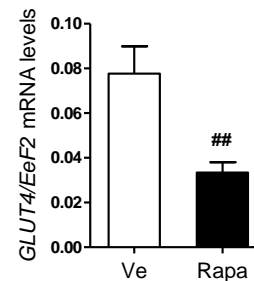

Supplement: Figure S1 — Effect of rapamycin on adiponectin and leptin blood levels, on respiratory quotient and locomotor activity and expression of adipogenic factors. (A) Adiponectin and leptin blood levels (Rapa: ▪, Ve: □). (B) Respiratory quotient (Rapa: ▪, Ve: □) and (C) locomotor activity, measured over a 36-hour monitoring period (16-week post-injection). (C) Real-time quantitative PCR (RT-qPCR) analysis of the VWAT of Ve- or Rapa-treated mice, after 22 injections (Rapa: ▪, Ve: □): Expression levels of PPARγ, C/EBPa, SREBP-1c, FAS, leptin and GLUT4. Data are expressed as mean ± S.E.M. of 8 to 10 mice per group. # p<0.05, ## p<0.01, ### p<0.001 #### p<0.0001. (PDF) [file pone.0092684.s001.pdf]

**A**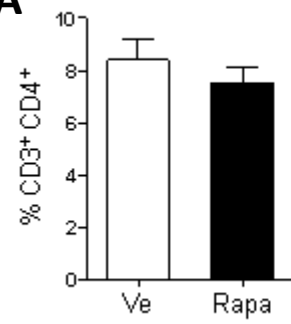**B**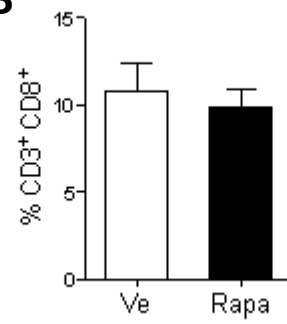**C**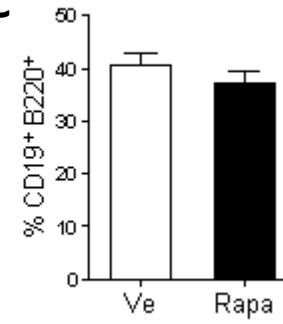

Supplement: Figure S2 — Effect of rapamycin on blood lymphocyte T CD4+, CD8+ and B cells. (A) CD3+ CD4+ T-cells, (B) CD3+ CD8+ T-cells, and (C) CD19+ B220+ B-cells were analyzed in the blood by flow cytometry (Rapa: ▪, Ve: □). Results are expressed as percentage of live cells. Data expressed as mean ± S.E.M. of 8 mice per group. (PDF) [file pone.0092684.s002.pdf]

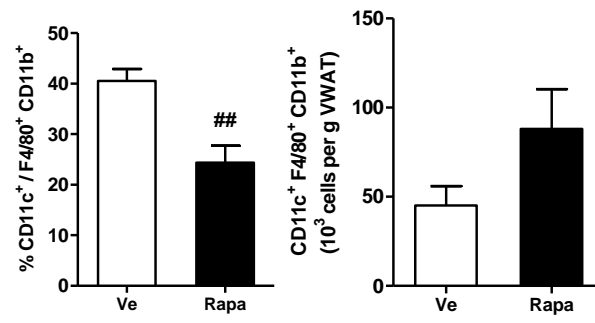

Supplement: Figure S3 — Effect of rapamycin on CD11b+ F4/80+ CD11c+ subset in adipose tissue stromal vascular fraction (SVF) (22 weeks post-injection). Adipose tissue CD11c-expressing macrophages were analyzed by flow cytometry. Results are expressed as percentage of CD11b+ F4/80+ cells (left panel) or as a cell number (×103)/VWAT mass (g) (right panel) (Rapa: ▪, Ve: □). (PDF) [file pone.0092684.s003.pdf]

**A**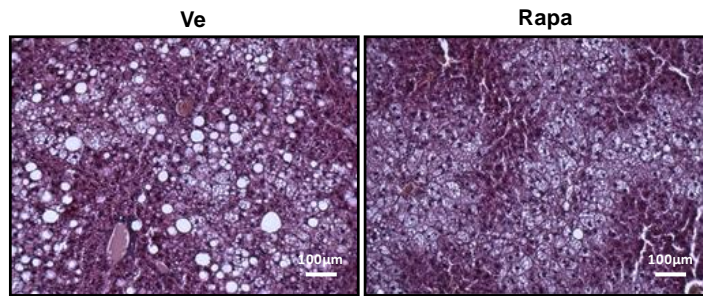**B**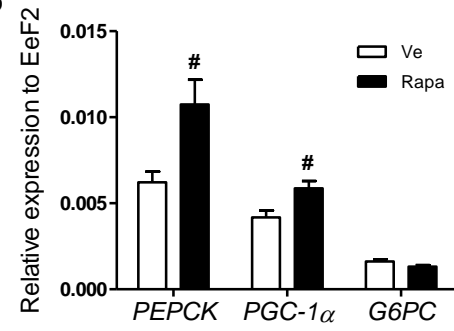

Supplement: Figure S4 — Liver histology and gluconeogenesis-related gene expression. (A) Representative sections of H&E-stained liver (Scale bars represent 100 µm). (B) Real-time quantitative PCR (RT-qPCR) analysis of the liver, after 22 injections (Rapa: ▪, Ve: □): Expression levels of the gluconeogenesis-related genes PEPCK, PGC-1α and G6PC. Data are expressed as mean ± S.E.M. of 8 to 10 mice per group. # p<0.05. (PDF) [file pone.0092684.s004.pdf]
