# Supplementary material for: Beneficial Metabolic Effects of Rapamycin Are Associated with Enhanced Regulatory Cells in Diet-Induced Obese Mice
Source: PLoS One. 2014 Apr 7;9(4):e92684. doi: 10.1371/journal.pone.0092684 (PMC3977858; doi:10.1371/journal.pone.0092684)
Supplement: Materials and Methods S1 — Glycerol, triglycerides, non-esterified fatty acids (NEFAs) and Sirolimus blood levels. Glycerol, triglycerides, non-esterified fatty acids (NEFAs) and Sirolimus blood levels were quantified using, respectively, colorimetric and chemiluminescence assays. (DOC) [file pone.0092684.s005.doc]

**Glycerol, triglycerides and non-esterified fatty acids (NEFAs) blood levels.**

Glycerol, triglycerides and NEFAs levels were determined in the sera of 12-hours fasted mice (at sacrifice) using colorimetric assays (respectively, Serum Triglycerides Determination Kit, Sigma-Aldrich and Free Fatty Acid Quantification Kit; BioVision, Mountain View, CA, USA).

**Sirolimus blood levels.**

After 16 weeks of rapamycin injections, blood samples were used to measure rapamycin levels using the Sirolimus chemiluminescence magnetic microparticle immunoassay (CMIA) on the Architect-i2000 system (Abbott, IL, USA).
