# Supplementary material for: Beneficial Metabolic Effects of Rapamycin Are Associated with Enhanced Regulatory Cells in Diet-Induced Obese Mice
Source: PLoS One. 2014 Apr 7;9(4):e92684. doi: 10.1371/journal.pone.0092684 (PMC3977858; doi:10.1371/journal.pone.0092684)
Supplement: Table S1 — Antibodies used for FACS. Primary antibodies used for FACS analysis (clones, dilution, origin). (PDF) [file pone.0092684.s006.pdf]

FACS antibodies	Dilution	Origine
F4/80 PE-Cy7 (BM8)	1:400	123113-Biolegend, San Diego, CA, USA
Ly6G APC (1A8)	1:1200	127613-Biolegend, San Diego, CA, USA
Ly6C Pacific Blue (HK1.4)	1:1600	128013-Biolegend, San Diego, CA, USA
CD11b PE (M1/70)	1:200	101225-Biolegend, San Diego, CA, USA
CD11c APC/Cy7 (N418)	1:100	117323-Biolegend, San Diego, CA, USA
CD3 PE-CF594 (145-2C11)	1:200	562286-BD Biosciences
CD4 Brillant Violet 605 (RM4-5)	1:200	100547-Biolegend, San Diego, CA, USA
CD8a APC-eF780 (53-6.7)	1 :200	17-0081-81-eBioscience, San Diego, CA, USA
FoxP3 PE (FJK-16s)	1:100	25-5773-80-eBioscience, San Diego, CA, USA
B220 BD V500 (RA3-6B2)	1:100	562290-BD Biosciences
CD19 Alexa Fluor 700 (6D5)	1:400	115527-Biolegend, San Diego, CA, USA
