# Supplementary material for: Beneficial Metabolic Effects of Rapamycin Are Associated with Enhanced Regulatory Cells in Diet-Induced Obese Mice
Source: PLoS One. 2014 Apr 7;9(4):e92684. doi: 10.1371/journal.pone.0092684 (PMC3977858; doi:10.1371/journal.pone.0092684)
Supplement: Table S2 — Antibodies used for western-blot. Primary antibodies used for western blot analysis (dilution, origin). (PDF) [file pone.0092684.s007.pdf]

Western blot antibodies	Dilution	Origine
rabbit polyclonal anti-total AKT	1:1000	#9272, Cell Signaling
rabbit polyclonal anti-phospho Ser473 AKT	1:1000	#9271, Cell Signaling
rabbit monoclonal anti-total P70 S6 kinase (49D7)	1:1000	#2708, Cell Signaling
rabbit polyclonal anti-phospho Thr389 P70 S6 kinase	1:800	#9205, Cell Signaling
rabbit polyclonal anti-phospho Ser612 IRS1	1:500	#2386, Cell Signaling
rabbit polyclonal anti-IRS1	1:500	Sc-560, Santa Cruz
rabbit polyclonal anti-IRS2	1:500	Sc-8299, Santa Cruz
