# Supplementary material for: Beneficial Metabolic Effects of Rapamycin Are Associated with Enhanced Regulatory Cells in Diet-Induced Obese Mice
Source: PLoS One. 2014 Apr 7;9(4):e92684. doi: 10.1371/journal.pone.0092684 (PMC3977858; doi:10.1371/journal.pone.0092684)
Supplement: Table S3 — Ingenuity Pathway Analysis (IPA) of deregulated genes in Rapamycin VWAT. Top enriched pathways in top enriched biological functions: Immune cell trafficking and Inflammatory response (p values, predicted activation state, # molecules). (PDF) [file pone.0092684.s008.pdf]

Top 5 enriched pathways in Immune Cell Trafficking

	p-Value	Predicted Activation State	# Molecules
Cell movement of myeloid cells	5,54E-12	Increased	54
Cell movement of phagocytes	3,39E-11	Increased	52
Cell movement of granulocytes	6,00E-08	Increased	38
Cell movement of neutrophils	5,67E-06	Increased	28
Recruitment of phagocytes	6,69E-06	Increased	25

Top 5 enriched pathways in Inflammatory Response

	p-Value	Predicted Activation State	# Molecules
Immune response	3.20E-20	Increased	144
Inflammatory response	7.69E-16	Increased	70
Cell movement of phagocytes	3.39E-11	Increased	52
Chemotaxis of leukocytes	4.15E-11	Increased	34
Quantity of phagocytes	3.76E-10	None	53
