# Supplementary material for: Beneficial Metabolic Effects of Rapamycin Are Associated with Enhanced Regulatory Cells in Diet-Induced Obese Mice
Source: PLoS One. 2014 Apr 7;9(4):e92684. doi: 10.1371/journal.pone.0092684 (PMC3977858; doi:10.1371/journal.pone.0092684)
Supplement: Table S4 — Blood lipid profiles in rapamycin-treated mice. Triglycerides, glycerol and non-esterified fatty acids (NEFAs) blood levels (in mmol/l). Results are expressed as mean ± S.E.M. of 8 to 10 mice per group. # p<0.05. (PDF) [file pone.0092684.s009.pdf]

Mean ± S.E.M (n=8/10)	Ve	Rapa
**Triglycerides (mmol/l)**	1.5 ± 0.2	1.1 ± 0.2$^{\#}$
**Glycerol (mmol/l)**	7.3 ± 2.2	6.4 ± 1.9
**NEFA (mmol/l)**	1.2 ± 0.06	1.5 ± 0.3
